# Association between melanopsin gene polymorphism (I394T) and pupillary light reflex is dependent on light wavelength

**DOI:** 10.1186/1880-6805-32-16

**Published:** 2013-10-12

**Authors:** Sang-il Lee, Akiko Hida, Sei-ichi Tsujimura, Takeshi Morita, Kazuo Mishima, Shigekazu Higuchi

**Affiliations:** 1 Department of Human Science, Faculty of Design, Kyushu University, 4-9-1 Shiobaru, Minami-ku, Fukuoka 815-8540, Japan; 2 Department of Psychophysiology, National Institute of Mental Health, National Center of Neurology and Psychiatry, 4-1-1 Ogawa-Higashi, Kodaira, Tokyo 187-8553, Japan; 3 Department of Information Science and Biomedical Engineering, Kagoshima University, 1-21-40 Korimoto Kagoshima, Kagoshima 890-0065, Japan; 4 Department of Living Environmental Science, Fukuoka women’s University, 1-1-1, Kasumigaoka, Higashi-ku, Fukuoka 813-8529, Japan

**Keywords:** Genotype, Human, Intrinsically photosensitive retinal ganglion cells, Melanopsin gene (*OPN4*), Non-image-forming responses, Pupillary light reflex, Single nucleotide polymorphism, Steady-state pupil response, Monochromatic light

## Abstract

**Background:**

Our aim was to determine the association between melanopsin gene polymorphism and pupillary light reflex under diverse photic conditions, including different intensities and wavelengths.

**Methods:**

A total of 195 visually corrected subjects volunteered for investigation of the melanopsin gene of single nucleotide polymorphism (SNP) of rs1079610 (I394T). The genotype groups were TT (*n* = 126), TC (*n* = 55), and CC (*n* = 8), and 75 of the subjects, including subjects with TT (*n* = 34), TC (*n* = 33), and CC (*n* = 8) participated in our experiment. Three monochromatic lights with peak wavelengths of 465 nm (blue), 536 nm (green), and 632 nm (red) were prepared, and each light was projected to the subjects with five intensities, 12, 13, 14, 14.5 and 15 log photons/(cm^2^ s), for one minute. The pupil size of the left eye was measured under each light condition after a 1-minute adaptation.

**Results:**

The pupils of the TC + CC genotypes (*n* = 38) were significantly smaller than those of the TT genotype (*n* = 31) under a blue (463 nm) light condition with 15 log photons/(cm^2^ s) (*P* < 0.05). In contrast, there were no significant differences under green (536 nm) and red (632 nm) light conditions. Conversely, relative pupil constrictions of the TC + CC genotypes were greater than those of the TT genotype under both blue and green conditions with high intensities (14.5 and 15 log photons/(cm^2^ s)). In contrast, there were no significant differences between genotype groups in pupil size and relative pupilloconstriction under the red light conditions.

**Conclusions:**

Our findings suggest that the melanopsin gene polymorphism (I394T) functionally interacts with pupillary light reflex, depending on light intensity and, particularly, wavelength, and that under a light condition fulfilling both high intensity and short wavelength, the pupillary light response of subjects with the C allele (TC + CC) is more sensitive to light than that of subjects with the TT genotype.

## Background

In mammals, a small subset of retinal ganglion cells express the photopigment melanopsin, and they are intrinsically photosensitive (hence, intrinsically photosensitive retinal ganglion cells, ipRGCs) [1-3]. Parallel studies have been carried out to identify the functional roles of melanopsin or ipRGCs using transgenic mice, such as mice lacking rods or cones [4,5] and mice lacking melanopsin [6,7]. We now know that ipRGCs transmit photic irradiance information to the suprachiasmatic nuclei, intergeniculate leaflet, and olivary pretectal nucleus through the retinohypothalamic tract [8,9] and that they play important roles in non-image-forming responses, including circadian photoentrainment [6,10], pupillary light reflex (PLR) [11,12], and other behavioral and physiological functions [13-15].

In human beings, there are large inter-individual phenotypic variations in the non-image-forming effects of light [16-18]. For instance, it is known that dark- and light-adapted pupil sizes in normal healthy subjects have large inter-individual differences [17,19,20]. What causes these inter-individual variations? According to the database of the International HapMap Project, in human beings, there are some genetic variations in the melanopsin (*OPN4*) gene. Higuchi *et al*. [21] attempted to demonstrate the functional differences of *OPN4* gene polymorphism (I394T) by measuring steady-state pupil response during exposure to light, and they revealed that there is a functional connection between *OPN4* gene polymorphism and pupillary light response. However, the spectral sensitivity of melanopsin was not considered in their study. Melanopsin is a vitamin A-based opsin, and all of the opsin and vitamin A-based photoreceptors have characteristic spectral sensitivities [22]. Parallel studies have shown the peak sensitivity of melanopsin in other mammals to be around 480 nm [23,24], unlike rods (*λ*_max_ 498 nm) and short, medium, and long-wavelength cones (*λ*_max_ 420, 534, and 563 nm) [25], and consistent with human melanopsin *λ*_max_[11,26]. Thus, we hypothesized, first, that functional differences of the *OPN4* gene polymorphism in PLR would be apparent at a short-wavelength light but not at a long-wavelength light related to M- and L-cone excitation and, second, that these differences depend on light intensity, as suggested by our previous study [21]. Hence, the aim of this study was to determine whether *OPN4* gene polymorphism in a young Japanese population is associated with steady-state pupil size during exposure to light of various wavelengths and intensities.

## Methods

### Subjects

Prior to the experiment, 195 Japanese university students were gathered to investigate *OPN4* gene polymorphism. This recruitment was totally different from that in our previous study [21]. All subjects had normal color vision, as tested by the Ishihara color vision test. Scalp hairs were used to extract genomic DNA samples, to genotype the single nucleotide polymorphism (SNP) of rs1079610 (I394T) located in the coding region. Genomic DNA was extracted from a hair using a DNA Extractor FM Kit (Wako Pure Chemical Industries, Ltd., Osaka, Japan). The genotype groups were classified as TT, TC, or CC, and the numbers of subjects in those groups were 126, 55, and 8, respectively (six being undetermined). The T and C allele frequencies of I394T were 81.2% and 18.8%, respectively. Genotype frequency of I394T was consistent with the Hardy-Weinberg equilibrium (*χ*^2^ = 0.40, ns). Subjects gave written informed consent for participation in the study, which was approved by the ethical committee of Kyushu University and the ethics committee of the National Center of Neurology and Psychiatry.

Seventy-five subjects, including subjects with TT (*n* = 34, 16 men and 18 women; 20.79 ± 2.0 years old), subjects with TC (*n* = 33, 16 men and 17 women; 21.03 ± 2.3 years old), and subjects with CC (*n* = 8, one man and seven women; 21.33 ± 1.7 years old) volunteered for the study and participated in all of the experiments. Exclusion criteria included medication or drug consumption and shift work.

### Experimental conditions

**Figure 1:**
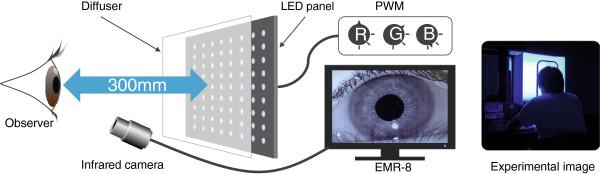
**Set-up for the experiments.** A total of 225 modules of a 3-in-1 type (RGB) LED on a square panel (350 × 350 mm) were prepared, and the intensity of lights was controlled by analog voltage-width modulation. The diffuser, located in front of the light device, was 300 mm from subject’s eyes. Pupil diameter was measured in the left eye using an EMR-8 eye-tracking system with an infrared camera (Nac Image Technology, Inc., Japan).

A total of 225 modules of a three-in-one type (red-green-blue, RGB) light-emitting diode (LED) on a square panel (350 × 350 mm) were prepared for exposure of subjects to monochromatic light. The intensity of lights was controlled by analog voltage-width modulation. A diffuser, located in front of the light device, was arranged 300 mm from subject’s eyes (Figure 1). To control the range of subject’s eye movement during the measurement, we marked five points forming a cross.

The peak wavelengths of each color condition were measured by an illuminance spectroradiometer (CL-500A, KONICA MINOLTA INC., Japan) in the vertical direction from the height of the subject’s eyes. The peak wavelengths were 632 nm (red), 536 nm (green), and 465 nm (blue) with half-bandwidths of 30 to 45 nm. Five intensity conditions, including 12, 13, 14, 14.5 and 15 log photons/(cm^2^ s), were modulated at each color condition equally. Table 1 shows the illuminance (lx) and irradiance (μW/cm^2^) levels of the respective light conditions.

**Table 1:** **Illuminance (lux) and irradiance (μW/cm**^**2**^**) levels of each light condition**

	**Blue**		**Green**		**Red**	
**Intensity log photons/(cm**^**2 **^**s)**	**lux**	**μW/cm**^**2**^	**lux**	**μW/cm**^**2**^	**lux**	**μW/cm**^**2**^
**12**	0.28	0.35	1.95	0.33	0.54	0.28
**13**	2.75	3.52	19.90	3.04	6.27	3.25
**14**	24.96	33.92	167.14	29.56	53.89	28.01
**14.5**	72.62	104.42	525.22	97.74	158.28	83.58
**15**	208.33	318.37	1391.48	285.49	505.54	272.59

### Procedure

The blue, green, and red stimuli were presented in separate sessions, consisting of five intensities. Dark adaptation (20 minutes) preceded each run, and test stimuli were presented in the dark for one minute in stages from low-intensity to strong-intensity. Pupil diameter was measured in the left eye using an EMR-8 eye-tracking system with an infrared camera (Nac Image Technology, Inc., Japan). After adaptation to each test stimulus, the steady-state pupil size was measured two or three times for 5 seconds with a sample rate of 60 Hz. Test stimuli were maintained during the measurements. The head of each subject was fastened to a chin rest during the measurements.

It is suggested that PLR is modulated by an endogenous circadian clock (24 hours) [27]. To minimize these circadian effects on PLR, experiments were performed during the daytime between 10 am and 5 pm and the time of each run was counterbalanced between *OPN4* genotypes.

### Data analysis

Six subjects (TT = 3, TC = 2, CC = 1) were excluded, owing to outlier or unstable data. Hence, data from a total of 69 subjects (TT = 31, TC + CC = 38) were used for statistical analysis. In our comparison between genotype groups (TT, TC, and CC), we treated TC and CC as one group, since our previous study showed that pupillary light responses of subjects who were homozygous (TT genotype) were significantly different from those of subjects who were heterozygous (TC genotype) and homozygous (CC genotype) and that there was no significant difference between TC and CC genotypes in PLR [21]. Thus, we focused on determining what differences are caused by the C allele and verifying the reproducibility of results of our previous study.

The steady-state pupil data in all experimental conditions were analyzed using a repeated-measures three-way analysis of variance (ANOVA) (IBM^©^ SPSS^©^ version 21) with genotype (TT, TC + CC) as between-subject factor and light conditions (color and intensity) as independent variables. Bonferroni post-hoc comparisons were conducted when main effects or interactions were present. Also, comparisons of pupil responses in all experimental conditions between the two genotype groups were performed using a two-sided, independent-sample Student’s *t* test. A *P* value less than 0.05 was considered statistically significant.

## Results

### *OPN4* polymorphism and pupil size

**Figure 2:**
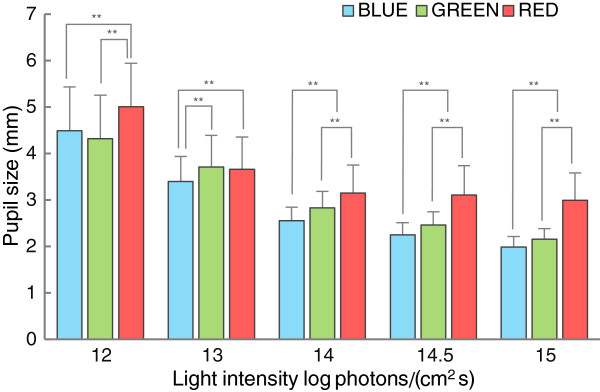
**Pupil sizes (mean + standard deviation) of all subjects under five intensities of blue light (blue bars), green light (green bars), and red light (red bars).** There were main effects of color (*P* < 0.001) and intensity (*P* < 0.001), and there was an interaction between color and intensity (*P* < 0.001). Pupil sizes under blue conditions were significantly smaller than those under the other color conditions. Pupil sizes under green conditions, except under the 13-green condition, were significantly smaller than those under red conditions. **P < 0.01.

Before the comparison between *OPN4* polymorphisms, it was important to confirm that the pupillary responses in this study were driven by ipRGCs. Figure 2 shows the pupil sizes of all subjects in all of the test conditions. In the analysis of pupil sizes of all subjects, there were main effects in both color (*F* = 197.394; *P* < 0.001) and intensity (*F* = 1532.19; *P* < 0.001) and there was an interaction between color and intensity (*F* = 118.961; *P* < 0.01) (Figure 2). The pupil sizes under blue conditions were significantly smaller than those under red conditions in all intensities. Similarly, the pupil sizes under green conditions were, except for 13 log photons/cm^2^ s, also smaller than those under red conditions. In the comparison between blue and green conditions, the pupil sizes in blue conditions were smaller than those in green conditions for all of the intensities except for the 12 log photons/cm^2^ s condition.

**Figure 3:**
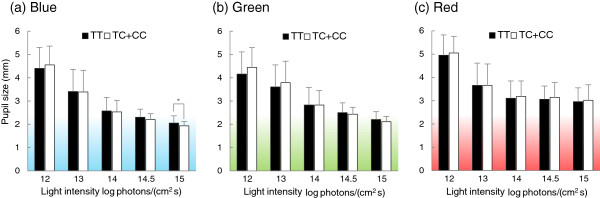
**Comparison of pupil sizes (mean + standard deviation) between TT (*n *= 31; black bars) and TC + CC (*n *= 38; white bars) genotypes. (a)** Blue conditions, **(b)** green conditions, **(c)** red conditions. Pupil sizes of TC + CC were significantly smaller than those of TT under the 15-blue condition, but there were no significant differences under green and red conditions. *P < 0.05.

Next (Figure 3), the repeated-measures three-way ANOVA (three levels of color × five levels of intensity × two groups of genotype) showed an interaction between color, intensity, and genotype (*F* = 3.702; *P* < 0.05), although the main effects of genotype were not significant. Results of the Student’s *t* test between the two groups of genotype showed that there was a significant difference only under the blue condition of 15 log photons/(cm^2^ s), and the pupil size of TC + CC genotypes was significantly smaller than that of TT genotype (*P* < 0.05). There were no significant differences between genotypes under green and red conditions.

### *OPN4* polymorphism and relative pupil constriction

**Figure 4:**
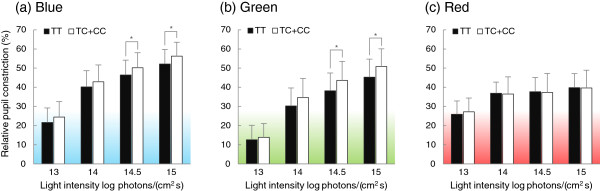
**Comparison of relative pupil constrictions (mean + standard deviation), based on 12 log photons/(cm**^**2 **^**s), between TT (*n *= 31; black bars) and TC + CC (*n *= 38; white bars) genotypes. (a)** Blue conditions, **(b)** Green conditions, **(c)** Red conditions. There were significant differences between TT and TC + CC under both blue and green conditions, but there was no difference under red conditions. *P < 0.05.

The relative pupil constriction was calculated as the percentage of pupil area under 13 to 15 log photons/(cm^2^ s) relative to the pupil area under 12 log photons/(cm^2^ s) of each color condition. In the relative pupil constriction (Figure 4), there were main effects in both color (*F* = 42.171; *P* < 0.001) and intensity (*F* = 2100.906; *P* < 0.001) and there was an interaction between color, intensity, and genotype (*F* = 3.901; *P* < 0.01). The Student’s *t* test showed that the relative pupilloconstriction of the TC + CC genotypes was larger than that of the TT genotype under the blue conditions of 14.5 and 15 log photons/(cm^2^ s) (*P* < 0.05), and the same results were obtained under the green conditions of 14.5 and 15 log photons/(cm^2^ s) (*P* < 0.05). However, there were no significant differences between genotypes under the red conditions.

## Discussion

The aim of this study was to determine the functional differences of the *OPN4* polymorphism (I394T) in PLR by addressing the relationship with light intensity and particularly light wavelength. First of all, it is important to confirm that the pupillary responses to the test stimuli were driven by ipRGCs. Several studies have demonstrated that the spectral characteristics of human ipRGCs have *λ*_max_ = 480 nm [11,26,28-30]. It has also been reported that melanopsin, included in ipRGCs, is the primary photopigment in driving pupillary response to high-irradiance light [31,32]. Consistent with those findings, our results showed that pupil sizes became smaller as the wavelength of light became closer to blue light (465 nm) and that pupil sizes decreased continuously with increasing intensity in both blue and green conditions. In contrast, there was no notable change in pupil size under red light conditions of 13 to 15 log photons/(cm^2^ s), indicating that ipRGCs were not triggered by red conditions. Hence, the experimental data obtained in the present study are considered to be a result of the excitement of ipRGCs.

In the comparison of pupil sizes between genotypes, a significant difference between TT and TC + CC genotypes was found solely under the blue light condition of 15 log photons/(cm^2^ s), and the pupil size of TC + CC genotypes was smaller than that of the TT genotype. Similarly, in our previous study, we found that *OPN4* gene I394T differences in PLR were apparent under a high-illuminance light condition [21]. The results of the present study, obtained using a new sample population, strongly support the results of our previous study. A notable result, however, is that a pupil size difference between the genotype groups only appeared under blue light. This seems to be a consequence of the spectral sensitivity of ipRGCs showing a peak at a short wavelength, as mentioned. Given that an association between genotypes and pupil size was found under a condition satisfying high intensity and short wavelength, our findings strongly suggest a causal relationship between I394T and PLR.

Moreover, significant differences between genotypes in relative pupillary constriction were found not only under blue light of high intensity, but also under green light of high intensity. Action spectra of ipRGCs [11] and results of several studies [33,34] have shown that green regions of light are effective for driving melanopsin, although melanopsin excitement is greater under blue than green light exposure. In this respect, our findings indicate that the melanopsin sensitivity of the TC + CC genotype was larger than that of the TT genotype when melanopsin was strongly stimulated.

It is notable that under the red light conditions, there was no significant difference between genotype groups in pupil size and pupil constriction, regardless of photic intensity. This is consistent with our hypothesis and supports our findings for genotype differences in PLR, because the red lights (λ_peak_ 632 nm) used in our study were expected to be correlated with activation of M- and L- cones (λ_max_ 534 and 563 nm) [25], not melanopsin. In support of this, human beings lacking the outer retina (i.e., blind but with normal melanopsin) were barely able or unable to detect long-wave light [32,35]. Hence, we predicted that there would be no significant differences between genotypes under the red light conditions.

Interestingly, there are geographic or ethnic differences in allele frequency of I394T. According to the International HapMap Project, C allele frequencies of I394T are 34.2% in Europeans, 27.8% in Chinese, 17% in Japanese, and 14.2% in Nigerians. It is not clear what caused these allele frequency differences, but it is obvious that the C allele frequency in Europeans is larger than that in people living in lower-latitude regions. Given that I394T genotype groups with the C allele were more sensitive to high-intensity lights than were the TT genotype group in this study, it would be interesting to determine whether the C allele is associated with biological adaptation in a photic environment. In addition, there is evidence to suggest ethnic differences in seasonal affective disorder [36], which is assumed to be increased as a result of the short photoperiod in winter [37]. Roecklein *et al*. [38] showed that an SNP of the melanopsin gene (P10L) was associated with prevalence of seasonal affective disorder. Although this study indicates a functional connection between *OPN4* gene polymorphism and a non-image-forming process, there was not sufficient physiological evidence. In future work, the functional differences between *OPN4* gene polymorphisms, including I394T and P10L, should be examined with other ethnic groups.

In terms of a selection of experimental photic stimuli for exciting melanopsin, there are ongoing debatable problems. For instance, our results showed that genotype differences in pupil size did not always appear under high-intensity lights. There are some claims that established photometric measures are inappropriate for quantifying effective light exposure for melanopsin [13], and a new measurement named ‘melanopic illuminance’ (m-lux) has been suggested to predict the sensitivity of melanopsin to lights [39,40]. Measurement of melanopic illuminance might be helpful to explain our findings in this study or our future work more precisely.

We used steady-state pupil response in this study, but there is an efficient method to assess ipRGC-driven pupil photoresponses called the post-illumination pupillary response (PIPR) [11,41]. This is a response after light offset, which means it is unknown whether PIPR represents the ipRGC-driven pupillary response to continuous light exposure, namely a real light environment, that we focused on in this study.

We determined an association between PLR and *OPN4* genotype groups in this study, indicating that the melanopsin sensitivity could be different, depending on the genotype of I394T. However, we still do not know the functional differences of the *OPN4* polymorphism (I394T) in other non-image-forming processing and how much the genotype differences in PLR could influence other irradiance responses. For example, ipRGCs also interact with light-induced melatonin suppression in human beings [42,43], and it has been reported that pupil size is correlated with melatonin suppression [17]. Further, human circadian phase could be shifted by exposure to high-intensity light and short-wavelength light [44,45], suggesting involvement of ipRGCs in human sleep-wake patterns. In addition, researchers in the field of physiological anthropology, which concerns human environmental adaptation, have revealed the influence of light on human physiological responses [46-49]. To validate our findings, it is necessary to determine the relationship between I394T and such physiological responses.

## Conclusions

In conclusion, human melanopsin gene polymorphism I394T functionally interacts with PLR depending on light intensity and, particularly, wavelength. Our findings suggest that, under a light condition that strongly excites melanopsin (high intensity and short wavelength), the pupillary light response of C allele subjects (TC + CC) is more sensitive to light than TT subjects. Further, we found that green light of high intensity, even though it activates melanopsin less than blue light, is also effective in eliciting a functional *OPN4* genetic difference in PLR.

## Abbreviations

ANOVA: Analysis of variance; ipRGCs: Iintrinsically photosensitive retinal ganglion cells; LED: Light-emitting diode; PIPR: Post-illumination pupillary response; PLR: Pupillary light reflex; RGB: Red-green-blue; SNP: Single nucleotide polymorphism.

## Competing interests

The authors declare that they have no competing interests.

## Authors’ contributions

SL collected experimental data, performed statistical analysis and wrote the manuscript. SL and SH participated in the design of the study. SL and AH carried out the molecular genetic analysis. KM, ST and TM revised the manuscript. SH supervised the study and helped to draft the manuscript. All authors read and approved the final manuscript.
